# Renal transplant in a child with Bardet-Biedl syndrome: A rare cause of end-stage renal disease

**DOI:** 10.4103/0971-4065.57108

**Published:** 2009-07

**Authors:** A. K. Hooda, S. C. Karan, J. S. Bishnoi, A. Nandwani, T. Sinha

**Affiliations:** Department of Nephrology, Army Hospital (R&R), Delhi Cantt, New Delhi-110 010, India; 1 Department of Urology, Army Hospital (R&R), Delhi Cantt, New Delhi-110 010, India

**Keywords:** Chronic kidney disease, retinal renal syndrome, renal transplantation

## Abstract

Bardet-Biedl syndrome (BBS) is a rare cause of renal failure requiring renal replacement therapy. It is an autosomal recessive condition characterized by retinitis pigmentosa, postaxial polydactyly, central obesity, mental retardation, hypogonadism, and renal involvement. We report the first successful renal transplant in a case of BBS from India.

## Introduction

Bardet-Biedl syndrome (BBS) is a rare autosomal recessive disorder characterized by rod-cone dystrophy (retinitis pigmentosa), postaxial polydactyly, central obesity, mental retardation, hypogonadism, and renal involvement.[1] It represents a very rare indication for kidney transplantation. Only 11 cases have been reported from India of which only one case had documented end-stage renal disease (ESRD) requiring renal replacement therapy.[2] We report the first case from India who has undergone successful living-related renal transplantation.

## Case Report

A 12-year-old male child presented with the complaints of polyuria and polydipsia of two months duration in April 2007. There was no history of weight loss, anorexia, nausea, vomiting, dysuria, hematuria, graveluria, recurrent urinary tract infection, or nocturnal enuresis. His past history included poor school performance and diminished distant vision since his age of eight years. He was born out of a nonconsanguinous marriage as a full-term normal vaginal delivery. He had a monozygotic twin and both had polydactyly in all limbs. There was no history of any illness in the neonatal period but his twin died at the age of two months due to a severe respiratory infection. His developmental milestones were however delayed.

**Figure 1:**
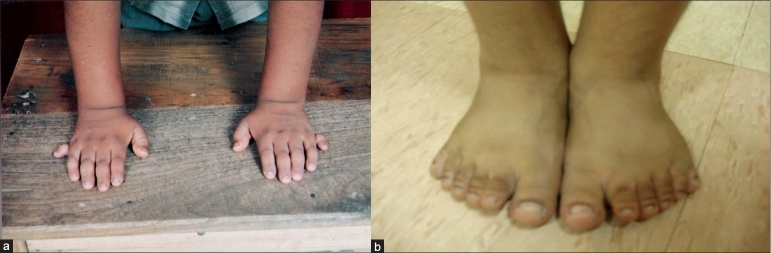
(a) Polydactyly in upper limbs, (b) Polydactyly in lower limbs

**Figure 2:**
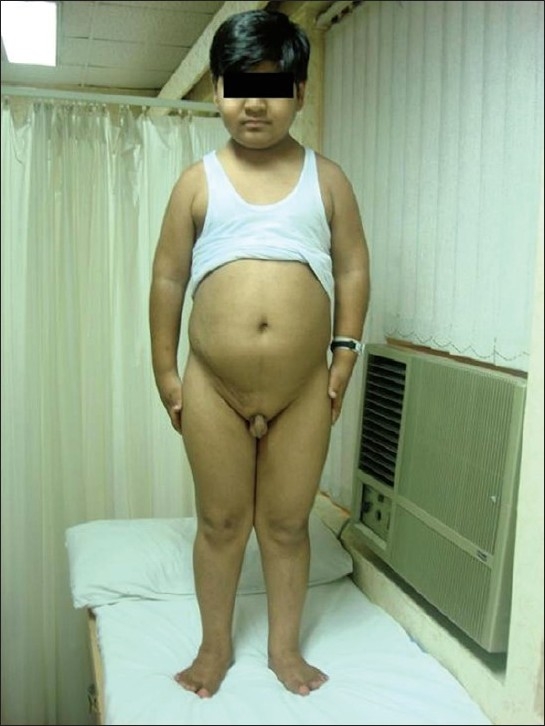
Obesity, hypogonadism, and scar of renal transplant surgery

Examination at presentation showed weight 44 kg (>75th percentile), height 128 cm (<5th percentile), BMI of 27.4 kg/m^2^, waist/hip ratio of 1.06, pulse 80/min, and BP 130/90 mmHg. He had pallor, no pedal edema and systemic examination was unremarkable. He had polydactyly in all four limbs [Figure 1] and features of hypogonadism with testicular volume of 2 ml (normal 10–12 ml) and micropenis (<2.5 cm) [Figure 2]. Ophthalmological examination showed bilateral impaired distant vision, normal anterior segment and lens, and fundus features of retinitis pigmentosa. Psychological evaluation revealed borderline mental retardation with an IQ of 71–75.

Investigations showed Hb 7.9 gm/dl, TLC 7000/cmm, 1 + proteinuria with no active urinary sediment, 24-hour urine protein of 240 mg, BUN 43 mg/dl, creatinine 4.3 mg/dl, serum sodium 143 mEq/l, serum potassium 3.8 mEq/l, serum total protein 8.1 gm/dl, albumin 3.9 gm/dl, calcium 9.4 mg/dl, phosphorus 3.9 mg/dl, serum alkaline phosphatase 486 U/l, and blood sugar F/PP 80/96 mg/dl. Ultrasound showed small kidneys (right 6.3 cm, left 6.7 cm) with loss of cortico-medullary differentiation, mild cortical irregularity, and normal PCS. DTPA scan showed bilateral impaired renal function (GFR right kidney 16.1 ml/min, left kidney 15.8 ml/min, and total GFR 31.9 ml/min). Echocardiography showed normal systolic function and no structural abnormality.

He was diagnosed as BBS with stage III chronic kidney disease and started on conservative management and underwent amputation of extra digits of hands. He progressed to ESRD in April 2008 with creatinine remaining consistently around 11 mg/dl and calculated GFR of 5.68 ml/min. He underwent preemptive renal transplant on May 02, 2008, his mother aged 38 years being the donor. His immunosuppression consisted of Tacrolimus 0.15 mg/kg, Mycophenolate mofetil 500 mg BD, and Prednisolone 0.5 mg/kg. He remained anuric in immediate postoperative period and no bruit was audible at graft site and color doppler showed graft vein thrombosis. He was re-explored and the operative findings confirmed graft vein thrombosis with extension of the thrombus in the external iliac vein. The graft was reperfused and reanastomosis was done after thrombectomy. His graft showed gradual return of function and he was discharged on 14^th^ postoperative day with a creatinine level of 1.2 mg/dl. At last follow-up on June 26, 2009 (14 months posttransplant), he was asymptomatic with urine output of >2 l/day, creatinine of 1.1 mg/dl, and DTPA scan GFR of graft kidney of 56.3 ml/min.

## Discussion

BBS is an autosomal recessive disorder characterized by obesity, retinal degeneration, polydactyly, hypogonadism, and mental retardation.[1] The detail biochemical mechanism that leads to BBS is still unclear. At this moment, 12 genes (BBS1 to BBS12) which are responsible for the disease when mutated, have been cloned. The gene products encoded by these BBS genes, called BBS proteins, are located in the basal body and cilia of the cell. Renal involvement in the form of various structural and functional abnormalities is common and renal insufficiency is noted in approximately 5–25%, with progression to ESRD in 4–10%. Renal failure is the most common cause of death in BBS.[3]

So far, a total of 11 cases have been reported from India, of which only one had ESRD.[2] Somwanshi *et al*. reported four cases with the typical phenotypic features of BBS in 1988.[4] Subsequently there have been another five case reports and all had normal renal function.[5,6] Prakash *et al*. reported another 30-year-old patient with BBS who had cysts in the left kidney with normal renal function.[7] Gupta *et al.* reported the first case of BBS from India with renal insufficiency (creatinine 3.0 mg/dl) in a 20-year-old female who had bilateral hypoplastic kidneys on ultrasound and also had multiple fractures, possibly related to renal osteodystrophy.[8] Rathi *et al*. described the first case from India with ESRD who was treated with continuous ambulatory peritoneal dialysis.[2]

The management of renal failure in BBS does not differ from that due to any other cause and all three modalities of long-term renal replacement therapy (RRT), i.e., hemodialysis, chronic peritoneal dialysis, and renal transplantation can be offered to these individuals. Still, it represents a very rare indication for kidney transplantation.[9,10] We have reported the first case of BBS with ESRD from India, who has undergone live-related renal transplantation. Our patient developed renal vein thrombosis in immediate posttransplant period which was successfully managed with reexploration, thrombectomy and reanastomosis. This complication is probably unrelated to the primary disease i.e., BBS and occurred as a technical complication.

To conclude, the diagnosis of BBS should be considered in patients with renal disease and the characteristic phenotype of retinitis pigmentosa, postaxial polydactyly, and central obesity. Renal transplantation is a viable option of RRT in these patients.
